# Laminin differentially regulates the stemness of type I and type II pericytes

**DOI:** 10.1186/s13287-017-0479-4

**Published:** 2017-02-07

**Authors:** Jyoti Gautam, Abhijit Nirwane, Yao Yao

**Affiliations:** College of Pharmacy, University of Minnesota, 1110 Kirby Drive, Duluth, MN 55812 USA

**Keywords:** Laminin, Pericytes, Proliferation, Differentiation

## Abstract

**Background:**

Laminin, a major basement membrane component that has direct contact with pericytes under physiological conditions, actively regulates the proliferation and differentiation/fate determination of pericytes. Recently, two types of pericytes (type I and type II) with different molecular markers and functions have been identified in skeletal muscles. Whether laminin differentially regulates the proliferation and differentiation of these two subpopulations remains unclear.

**Methods:**

Wild-type and pericytic laminin-deficient mice under Nestin-GFP background were used to determine if laminin differentially regulates the proliferation and differentiation of type I and type II pericytes. Specifically, type I and type II pericytes were isolated from these mice, and their proliferation and differentiation were examined in vitro. Moreover, in vivo studies were also performed.

**Results:**

We demonstrate that, although laminin inhibits the proliferation of both type I and type II pericytes in vitro, loss of laminin predominantly induces proliferation of type II pericytes in vivo. In addition, laminin negatively regulates the adipogenic differentiation of type I pericytes and positively regulates the myogenic differentiation of type II pericytes in vitro.

**Conclusions:**

Laminin differentially regulates the proliferation and differentiation of type I and type II pericytes.

**Electronic supplementary material:**

The online version of this article (doi:10.1186/s13287-017-0479-4) contains supplementary material, which is available to authorized users.

## Background

Satellite cells (postnatal muscle progenitor cells) are sandwiched between the basal lamina and myofiber plasma membrane in skeletal muscles [1]. Unlike satellite cells, various other cell populations, including fibro/adipogenic progenitors (FAPs) [2, 3], PW1^+/^Pax7^−^ interstitial cells (PICs) [4], mesenchymal stem cells (MSCs) [5–7], fibroblasts [8], and pericytes [9, 10], are found in the interstitial space of skeletal muscles. These populations are distinct from each other in their marker expression and differentiation abilities/functions. Specifically, FAPs are α7-integrin^−^Sca-1^+^PDGFRα^+^, and produce both fibroblasts and adipocytes [2, 3]. PICs are Sca-1^Med^CD34^+^PW1^+^Pax7^−^ and have myogenic potential [4]. MSCs express a wide range of stem cell markers and are able to generate adipose, bone, cartilage, and muscle [5–7, 11, 12]. Fibroblasts express ER-TR7, FSP1, and Tcf4, and are involved in tissue fibrosis [8]. Pericytes (perivascular cells covering capillaries in the vascular tree [13]) express a unique array of markers, including PDGFRβ, NG2, CD146, and CD13 [13]. It should be noted, however, that none of these markers are specific for pericytes since they are also expressed by other cell types, and their expression in pericytes is highly dependent on the developmental stages [13]. Accumulating evidence suggests that pericytes are multipotent cells. On one hand, pericytes are able to differentiate into myogenic cells via myogenesis to repair muscle injury [9, 10, 14–16]. On the other hand, pericytes can also differentiate into adipocytes and/or fibroblasts [14, 17–21], exacerbating muscle injury.

Recently, two subtypes (termed type I and type II) of pericytes with different molecular markers and differentiation abilities have been reported in skeletal muscles. Biochemically, both type I and type II pericytes express various classical pericyte markers, including PDGFRβ, NG2, and CD146 [14, 22]. It is the expression of Nestin, an intermediate filament protein, that differentiates these two subtypes of pericytes: type I (PDGFRβ^+^/NG2^+^/CD146^+^ and Nestin^−^) and type II (PDGFRβ^+^/NG2^+^/CD146^+^ and Nestin^+^) [14, 22, 23]. It is worth noting that “Nestin” here refers to transgene Nestin-GFP from the Nestin-GFP mice (see Methods), not endogenous Nestin. Since Nestin-GFP cells express low levels of endogenous Nestin in bone marrow [24], caution should be taken when using endogenous Nestin to distinguish type I and type II pericytes. Further studies conducted by Birbrair and colleagues elegantly demonstrate that type I pericytes differentiate into adipogenic and fibrogenic cells, contributing to muscle degeneration, whereas type II pericytes undergo myogenesis, promoting muscle regeneration [14, 23, 25, 26]. These data suggest that type I and type II pericytes are intrinsically different, and have distinct differentiation capability and fate.

The molecular mechanisms underlying pericyte differentiation and fate determination are not fully understood. In a previous study, we identified laminin as a critical regulator in the differentiation and fate determination of PDGFRβ^+^ pericytes [21]. Specifically, we found that laminin negatively regulates their proliferation, inhibits their adipogenic differentiation, and is indispensable for their myogenic differentiation [21]. Whether laminin differentially regulates the differentiation and fate determination of type I and type II pericytes, however, remains unclear. Using the Nestin-GFP and laminin conditional knockout mice, we report here that laminin inhibits the proliferation of both type I and type II pericytes in vitro. Type II pericytes, however, predominantly proliferate in vivo upon loss of laminin. In addition, laminin prevents adipogenic differentiation of type I pericytes, but is required for myogenic differentiation of type II pericytes. These data suggest that laminin inhibits the proliferation of type II pericytes, and has anti-adipogenic and pro-myogenic effects on type I and type II pericytes, respectively.

## Methods

### Animals

Pdgfrβ-Cre^+^ line [27] and laminin γ1 floxed (F/F) transgenic mice [28] were crossed to generate laminin-deficient PKO (F/F:Pdgfrβ-Cre^+^) mice. These mice were further crossed with Nestin-GFP mice [29, 30] to generate control (F/F:Nestin-GFP) and PKO (F/F:Pdgfrβ-Cre^+^:Nestin-GFP) mice under Nestin-GFP background. All mice used in this study were in a C57BL/6-FVB-BALB/cBy mixed background. Both males and females were used in this study. All mice were maintained in the animal facility at University of Minnesota with free access to water and food.

### Muscle dissection and preparation

Mice were anesthetized and hindlimb muscles were carefully dissected and minced with scissors and blades. The minced muscle fragments were incubated with 0.2% (w/v) type-2 collagenase (Worthington, LS004176) in DMEM at 37 °C for 2 h with shaking, followed by 3 × 15 min incubations with 0.25% trypsin/EDTA at 37 °C. After trituration and centrifugation at 1500 rpm for 6 min, the supernatant was discarded and the pellet resuspended in RBC lysis buffer. After centrifugation, the pellet was resuspended in DMEM + 10% FBS and passed through a 40-μm cell strainer to remove aggregates. The resulting single cell solution was centrifuged again at 1500 rpm for 6 min and resuspended in sorting buffer (20 mM HEPES pH 7.0, 1 mM EDTA, 1% BSA in 1 × Ca/Mg^2+^-free PBS pH 7.0). The cells were then stained with different antibodies and subjected to FACS sorting.

### Cell sorting

Type I and type II pericytes were isolated from wild-type (WT) and PKO mice by FACS as described previously with minor modifications [9, 10, 14]. In brief, the single cell solution was stained with PDGFRβ-PE (eBioscience, 12-1402) on ice for 30 min. After extensive wash, DAPI was added, and PE^+^GFP^−^ (type I) and PE^+^GFP^+^ (type II) cells were sorted using the Sony SH800 sorter. Forward and back scatters (FSC and BSC) and DAPI were used to set a primary gate to exclude dead cells and small debris. Fluorescence minus one (FMO) control for PE was used to set the PDGFRβ-PE gating boundary. Cells dissociated from C57BL/6 mice (not labeled with Nestin-GFP) were used to set the Nestin-GFP gating boundary. The cells were sorted into culturing medium for in vitro experiments.

### Cell culture

FACS-isolated type I and type II pericytes were grown in pericyte medium/growth medium (ScienCell, 1201), myogenic differentiation medium (DMEM with 2% horse serum and 1% penicillin/streptomycin), or adipogenic differentiation medium (MesenCult™ Basal Medium supplemented with Adipogenic Stimulatory Supplement, STEMCELL, 05501 and 05503) at 37 °C with 5% CO_2_. Medium was changed every 2–3 days until analysis.

### In vitro proliferation assay

Proliferation was examined using the Click-iT Plus EdU Alexa Fluor 594 Imaging Kit (Life Technologies, C10639). Freshly isolated type I and type II pericytes were plated in uncoated or laminin-111 (Invitrogen, 23017-015, 5 μg/ml)-coated 24-well plates in pericyte medium. Two days after plating, Edu (8 μM) was added to the cells. After 24 h, the cells were fixed and Edu incorporation was analyzed according to the manufacturer’s instructions. For quantification, the percentage of Edu^+^ nuclei among all nuclei in one field was calculated. At least eight random fields per sample and five samples were used for quantification.

### In vitro adipogenic differentiation assay

Adipogenic differentiation was induced using adipogenic differentiation medium. Briefly, freshly isolated type I and type II pericytes were plated in uncoated or laminin-111 (5 μg/ml)-coated 24-well plates in pericyte medium. After 4 days, pericyte medium was replaced with adipogenic differentiation medium containing saline or laminin-111 (5 μg/ml). After 8 or 14 days, the cells were fixed and subjected to perilipin immunostaining, followed by Oil Red O/hematoxylin staining. Mean perilipin intensity per field was used to quantify the extent of adipogenesis. At least eight random fields per sample and four samples were used for quantification.

### In vitro myogenic differentiation assay

Myogenic differentiation was induced using myogenic differentiation medium. Briefly, freshly isolated type I and type II pericytes were plated in uncoated or laminin-111 (5 μg/ml)-coated 24-well plates in pericyte medium. After 4 days, pericyte medium was replaced with myogenic differentiation medium containing saline or laminin-111 (5 μg/ml). After 14 days, the cells were fixed and subjected to S-Myosin (DSHB, MF20, 1:100) immunostaining. Mean S-Myosin intensity per field was used to quantify the extent of myogenesis. At least eight random fields per sample and four samples were used for quantification.

### Immunohistochemistry and immunocytochemistry

Transverse muscle sections (10-μm thick) were cut using a cryostat. Sections of the muscles from one-third and two-thirds along the anterior/posterior axis were collected. Muscle sections or sorted cells were immunostained with anti-laminin γ1 (Abcam, AB3297, 1:200; NeoMarkers, RT-795-P0, 1:100), anti-PDGFRβ (eBioscience, 14-1402, 1:100), anti-S-Myosin (DSHB, MF-20, 1:100), anti-perilipin (Sigma, P1998, 1:500), anti-phospho-histone H3 (Millipore, 06-570, 1:500), or anti-Ki67 (Millipore, AB9260, 1:1000) antibodies overnight at 4 °C, followed by fluorescent secondary antibodies (Invitrogen) for 1 h at room temperature. After mounting, sections were examined and photographed with a LSM 710 confocal microscopy. For immunohistochemistry, five random fields from each section, two sections (one-third and two-thirds along the anterior/posterior axis) per sample, and five animals per condition were used for quantification. For immunocytochemistry, eight random fields from each experiment and four to five independent sorts/experiments were used for quantification.

### Histology

Oil Red O staining and hematoxylin staining were performed after immunocytochemistry, according to standard protocols. For Oil Red O staining, after the last step of perilipin immunocytochemistry, sections were washed in PBS and afterwards rinsed in 60% isopropanol. After incubating in Oil Red O (Sigma, O1391) working solution for 15 min, sections were rinsed in 60% isopropanol and water. Then, the sections were either mounted in mounting medium with DAPI (Vector Lab, H-1200) or stained in hematoxylin (Fisher Scientific, SH30-500D) for 2 min and then mounted in mounting medium (Vector Lab, H-1000). The percentage of Oil Red O^+^ area in each field was used to quantify the extent of adipogenesis. At least eight random fields per sample and four samples were used for quantification.

### Quantitative RT-PCR

RNA isolation was performed using Trizol reagent (Invitrogen), followed by RNeasy mini kits (Qiagen). RNA concentration was quantified using a ND1000 spectrophotometer (Nanodrop). Reverse transcription was performed using SuperScript III First-Strand Synthesis System (Invitrogen). Gene expression analysis was performed using SYBR Green PCR Master Mix (Qiagen) on a LightCycler 480 system (Roche). The qRT-PCR data were quantified using the comparative Ct method [31, 32]. Specifically, the Ct values for gene of interest (GOI) and the internal controls (IC) in WT and PKO samples were obtained. The relative expression change of GOI in PKO pericytes compared with WT pericytes is calculated using this equation: fold change = 2^–ΔΔCt^ = [(Ct-GOI – Ct-IC)_PKO_ – (Ct-GOI – Ct-IC)_wildtype_]. Each sample was performed in duplicate, and four independent/biological replicates were used for quantification. The primers used are listed below. C/EBPα: forward-CCCCTCAGTCCCTGTCTTTAGA, reverse-GGTGGAGGTGCAAAAAGCAA; C/EBPβ: forward-AAGCTGAGCGACGAGTACAAGA, reverse-GTCAGCTCCAGCACCTTGTG; C/EBPδ: forward-GCCCCAAAAGCCAGTAATTGT, reverse-ACAACAGGCCGTGCAGATC; KROX20: forward-CTGGGCAAAGGACCTTGATG, reverse-TCTTTTTGCTCCCCCATTTCT; KLF5: forward-GACATGCCCAGTTCGACAAA, reverse-CATGCCCTGGAACTGTTTCAT; FABP4: forward-CCCAACATGATCATCAGCGTAA, reverse-GTCGTCTGCGGTGATTTCATC; Pax7: forward-GGCCAAACTGCTGTTGATTACC, reverse-GTAGGCTTGTCCCGTTTCCA; MyoD: forward-GACCCAGGAACTGGGATATG, reverse-CGAAACACGGGTCATCATAG; Myf5: forward-TTCCCACCTGCTTCTCTGAAG, reverse-TCAAACTGGTCCCCAAACTCA; Mrf4: forward-CCCCCCTTTCCACCTAATCA, reverse-GACTTTCACTTGAGGTGGTGAGAA; Myog: forward-GACCCTACAGACGCCCACAA, reverse-CAATCTCAGTTGGGCATGGTT; S-Myosin: forward-AACAAGGACCCCCTGAATGAG, reverse-ACCACCGCCACCAGACTCT; GAPDH: forward-GGTGGAGCCAAAAGGGTCAT, reverse-GCATTGCTGACAATCTTGAGTGA; TBP: forward-CCCTTGTACCCTTCACCAAT, reverse-CAGCCAAGATTCACGGTAGA.

### Western blotting

Cells were lysed with RIPA buffer (50 mM Tris pH 7.4, 1% NP-40, 0.5% Na-deoxycholate, 1% SDS, 150 mM NaCl, 2 mM EDTA, 1 × protease inhibitor cocktail, and 1 × phosphatase inhibitor cocktail). Total protein levels were determined using the Bio-Rad protein assay kit, and equal amounts of proteins were loaded and separated on SDS-PAGE. After transferring to PVDF membrane (Millipore), proteins were detected using a standard immune-blotting technique. The following primary antibodies were used: anti-Pax7 (Aviva Systems Biology, ARP32742, 1:400), anti-Myf5 (Santa Cruz, sc-302, 1:200), anti-Myogenin (BD Biosciences, 556358, 1:250), anti-phospho-ERK1/2 (Cell Signaling, 4370, 1:1000), anti-ERK1/2 (Cell Signaling, 9107, 1:1000), anti-phospho-p38 (Cell Signaling, 9216, 1:1000), anti-p38 (Cell Signaling, 9212, 1:1000), anti-integrin α7 (Calbiochem, ST1637, 1:500), anti-gpihbp1 (Thermo Scientific, PA1-16976, 1:250), anti-GAPDH (Abcam, ab9484, 1:1000), and anti-β-actin (Sigma, A5441, 1:2000). Target proteins were visualized using SuperSignal West Pico Chemiluminescent Substrate (Pierce). The density of target protein bands was quantified using NIH ImageJ software. The expression of phospho-ERK1/2 and phospho-p38 was normalized to ERK1/2 and p38, respectively. The expression of all other proteins was normalized to GAPDH and/or β-actin.

### ELISA

The levels of BMP-2 and BMP-4 proteins synthesized by type I pericytes were determined by ELISA. Briefly, WT and PKO type I pericytes were plated in 24-well plates in the absence or presence of exogenous laminin-111 (5 μg/ml). The conditioned medium and cell lysates were collected after adipogenic differentiation for 8 days. BMP-2 and BMP-4 levels were determined by ELISA according to the manufacturer’s instructions (ls-f10947 and ls-f13543, LifeSpan Biosciences). BMP-2 and BMP-4 expression was normalized to total protein level.

### Statistics

Results are shown as mean ± SD. Student’s *t* test performed by SPSS Statistics was used to analyze differences between two groups (genotypes). Two-way ANOVA in SPSS Statistics was used for comparisons involving two factors (genotypes and treatments).

## Results

### Laminin is abrogated in both type I and type II pericytes in PKO mice

**Fig. 1:**
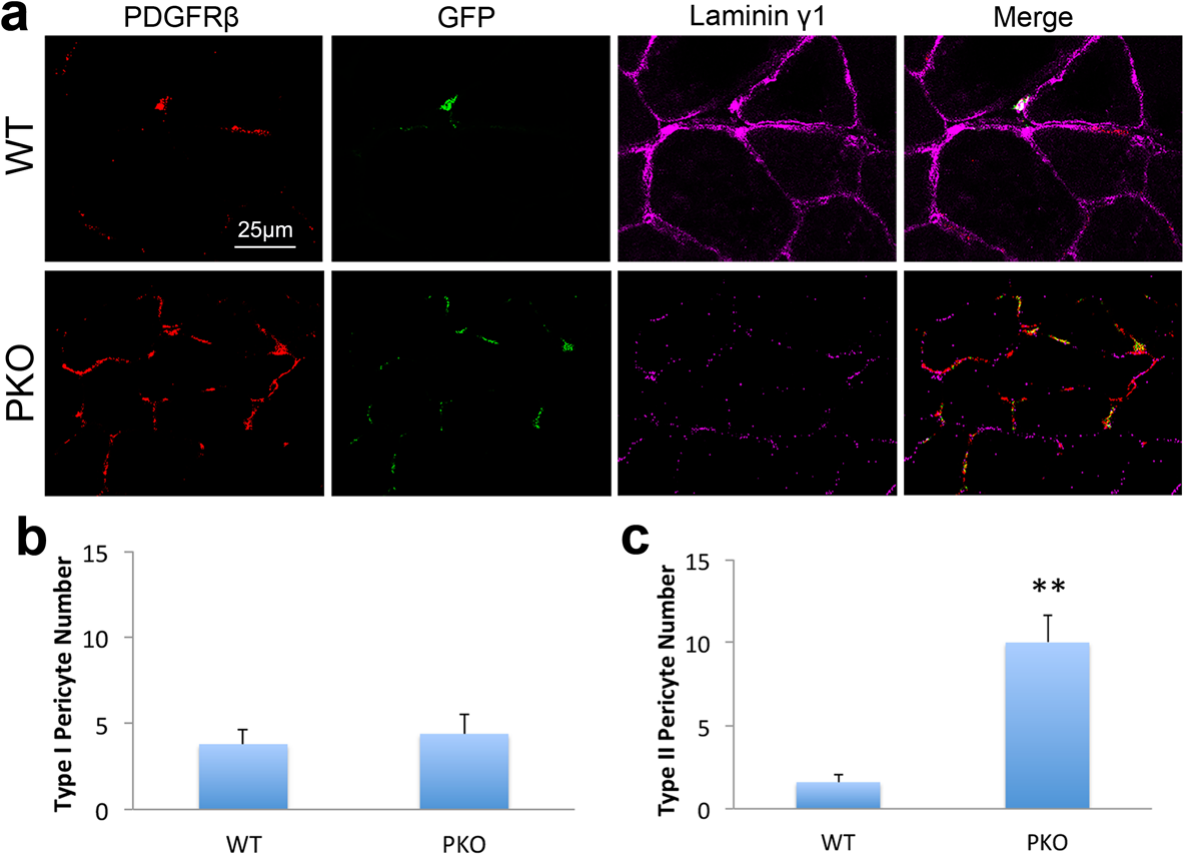
Both type I and type II pericytes are found in skeletal muscles. **a** Immunohistochemical analysis of PDGFRβ (*red*), GFP (*green*), and laminin γ1 (*magenta*) expression in wild-type (*WT*) controls and laminin γ1^flox/flox^:Pdgfrβ-Cre^+^(*PKO*) skeletal muscles. **b** and **c** Quantification of type I (**b**) and type II (**c**) pericyte number in WT and PKO muscles. *n* = 5. Data are shown as mean ± SD. **p < 0.01 versus WT. *Scale bar* = 25 μm

In a previous study [21], we generated a mouse line (PKO) with laminin deficiency in pericytes by crossing the laminin γ1^flox/flox^ (F/F) mice [28] with the Pdgfrβ-Cre^+^ transgenic line [27]. To investigate if laminin differentially regulates the proliferation and differentiation of type I and type II pericytes, we crossed these mice with Nestin-GFP transgenic line [29, 30] to generate control (WT) and PKO mice under Nestin-GFP background. Consistent with previous reports [14, 22, 23], both type I (PDGFRβ^+^Nestin^−^) and type II (PDGFRβ^+^Nestin^+^) pericytes were observed in skeletal muscles from WT mice (Fig. 1a). Both cell types were located in the interstitial space and expressed laminin (Fig. 1a). Consistent with our previous finding [21], laminin expression was significantly reduced and PDGFRβ expression increased in PKO muscles (Fig. 1a). Interestingly, GFP^+^ cells were also increased in PKO muscles, and most, if not all, GFP^+^ cells expressed PDGFRβ (Fig. 1a). Although no difference in type I pericyte number was observed (Fig. 1b), quantification revealed significantly more type II pericytes in PKO mice, compared to WT controls (Fig. 1c), indicating that loss of laminin preferably enhances type II pericyte number in vivo.

**Fig. 2:**
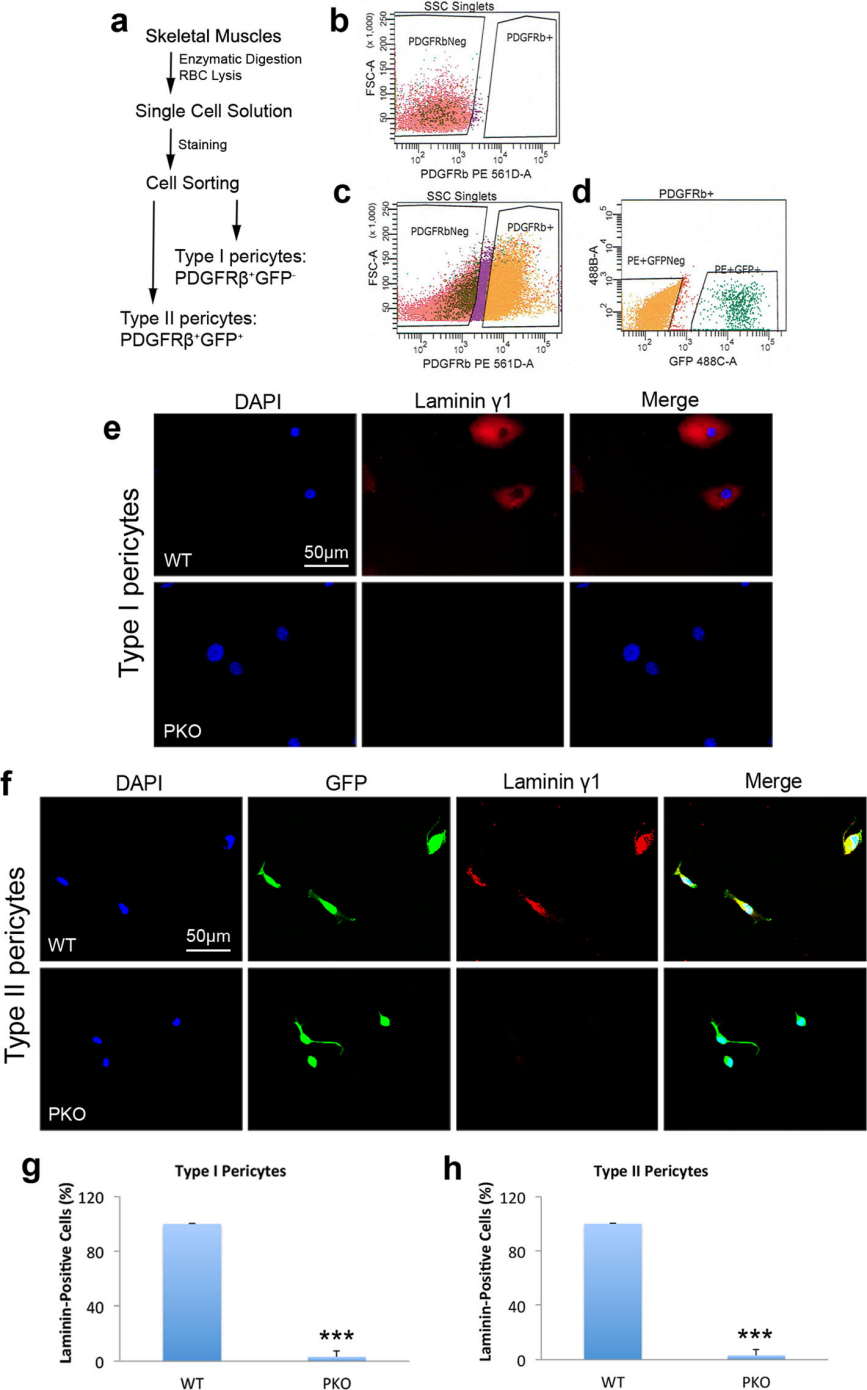
Laminin γ1 expression is abrogated in both type I and type II pericytes isolated from laminin γ1^flox/flox^:Pdgfrβ-Cre^+^(*PKO*) mice. **a** Diagram of type I and type II pericyte isolation procedure. **b** Representative plot of PDGFRβ-FMO control. **c**, **d** Representative plots showing sorting gates for PDGFRβ (**c**) and type I and type II pericytes (**d**). **e** Immunocytochemical analysis of laminin γ1 (*red*) expression in type I pericytes isolated from wild-type (*WT*) and PKO muscles. **f** Immunocytochemical analysis of laminin γ1 (*red*) and GFP (*green*) expression in type II pericytes isolated from WT and PKO muscles. **g**, **h** Quantitative data demonstrating the percentage of laminin-positive cells in type I (**g**) and type II (**h**) pericytes sorted from WT and PKO mice. *n* = 5. Data are shown as mean ± SD. ***p < 0.001 versus WT. *Scale bars* = 50 μm

In addition, laminin γ1 expression was absent in both PDGFRβ^+^Nestin^−^ and PDGFRβ^+^Nestin^+^ cells in PKO muscles (Fig. 1a), suggesting that laminin expression is diminished in both type I and type II pericytes in PKO mice. To further examine laminin expression in type I and type II pericytes, we isolated these cells from WT and PKO muscles by FACS using a well-established protocol [9, 10, 14, 21] (Fig. 2a). FMO control for PDGFRβ was used to set its gating (Fig. 2b and c). Sorted PDGFRβ^+^ cells were further separated based on GFP expression, and PDGFRβ^+^GFP^−^ and PDGFRβ^+^GFP^+^ populations were defined as type I and type II pericytes, respectively (Fig. 2d). Representative dot plots for PDGFRβ and GFP are shown in Additional file 1 (Figure S1). The purity of sorted pericytes was examined by immunocytochemistry against PDGFRβ and NG2, and found to be higher than 95%. Consistent with in vivo studies, laminin γ1 expression was detected in both type I and type II pericytes isolated from WT muscles, but was absent in both cell types from PKO muscles (Fig. 2e and f). Quantification showed that almost all WT pericytes (both type I and type II) examined in five independent experiments expressed laminin, while more than 95% of PKO pericytes (both type I and type II) failed to express laminin (Fig. 2g and h). These data again suggest that laminin expression is abrogated in both type I and type II pericytes in PKO mice.

### Loss of laminin preferentially induces type II pericyte proliferation in vivo

**Fig. 3:**
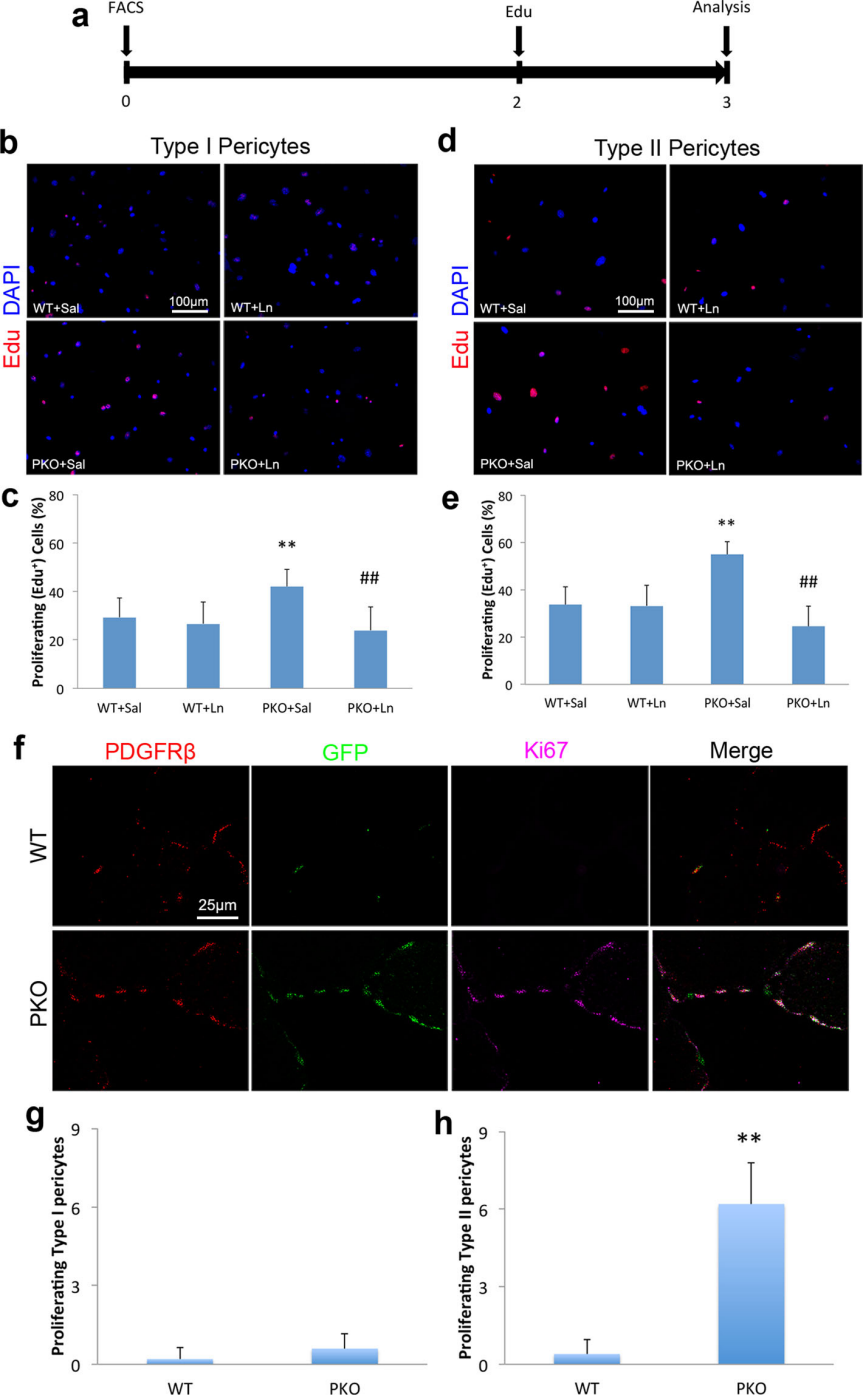
Laminin negatively regulates the proliferation of type I and type II pericytes. **a** Diagram of experimental design for Edu incorporation assay. **b** Immunocytochemical analysis of Edu (*red*) in type I pericytes isolated from wild-type (*WT*) and laminin γ1^flox/flox^:Pdgfrβ-Cre^+^(*PKO*) mice in the presence of saline (*Sal*) or laminin-111 (*Ln*). **c** Quantification of Edu incorporation in type I pericytes. *n* = 5. **d** Immunocytochemical analysis of Edu (*red*) in type II pericytes isolated from WT and PKO mice in the presence of Sal or Ln. **e** Quantification of Edu incorporation in type II pericytes. *n* = 5. **f** Immunohistochemical analysis of PDGFRβ (*red*), GFP (*green*), and Ki67 (*magenta*) expression in WT and PKO muscles. **g**, **h** Quantification of proliferating type I (**g**) and type II (**h**) pericyte number in WT and PKO mice. *n* = 5. Data are shown as mean ± SD. **p < 0.01 versus WT + Sal or WT; ^##^ *p* < 0.01 versus PKO + Sal. *Scale bars* = 100 μm in **b** and **d**, and 25 μm in **f**

In a previous study, we reported increased proliferation of laminin-deficient pericytes [21]. To investigate if the proliferation of type I and/or type II pericytes is regulated by laminin, we first performed in vitro proliferation assay. Type I and type II pericytes were isolated from WT and PKO mice by FACS as described above and their proliferation was examined by Edu incorporation assay 2 days post-isolation (Fig. 3a). Dramatically increased Edu^+^ cells were observed in PKO type I pericytes, compared to WT controls (Fig. 3b). Statistical analysis showed that there was a significant difference in the percentage of Edu^+^ cells between WT and PKO type I pericytes (Fig. 3c). Similar result was found for type II pericytes (Fig. 3d and e), suggesting that laminin negatively regulates the proliferation of both type I and type II pericytes in vitro. Additionally, exogenous laminin was able to substantially decrease the proliferation rate of both type I (Fig. 3b and c) and type II (Fig. 3d and e) PKO pericytes without affecting that of WT pericytes, indicating a reversible role of laminin in the proliferation of both type I and type II pericytes. In addition to Edu, an S-phase-specific marker [33], we also examined proliferation using phospho-Histone H3 (pH3), an M-phase-specific marker [34–36]. Similar results were observed; specifically, a higher percentage of pH3^+^ cells was found in PKO pericytes (both type I and type II), and exogenous laminin substantially reduced their proliferation to baseline levels (Additional file 1: Figure S2), again suggesting that laminin inhibits the proliferation of both type I and type II pericytes in vitro.

Next, we further examined the proliferation of pericytes in vivo by immunohistochemistry. Consistent with previous data [21], few proliferating (Ki67^+^) pericytes were observed in WT muscles (Fig. 3f). In PKO muscles, on the contrary, a large number of Ki67^+^ cells were identified (Fig. 3f). Surprisingly, these Ki67^+^ proliferating cells expressed both PDGFRβ and GFP (Fig. 3f). We then quantified the number of proliferating type I (Ki67^+^PDGFRβ^+^GFP^−^) and type II (Ki67^+^PDGFRβ^+^GFP^+^) pericytes in WT and PKO muscles. Although a comparable amount of proliferating type I pericytes was observed in WT and PKO mice (Fig. 3g), significantly more proliferating type II pericytes were detected in PKO muscles (Fig. 3h), suggesting that loss of laminin preferentially induces proliferation of type II pericytes in vivo. These data are consistent with the reported myogenic function of type II pericytes [14] and muscular dystrophic phenotype of the PKO mice [21].

**Fig. 4:**
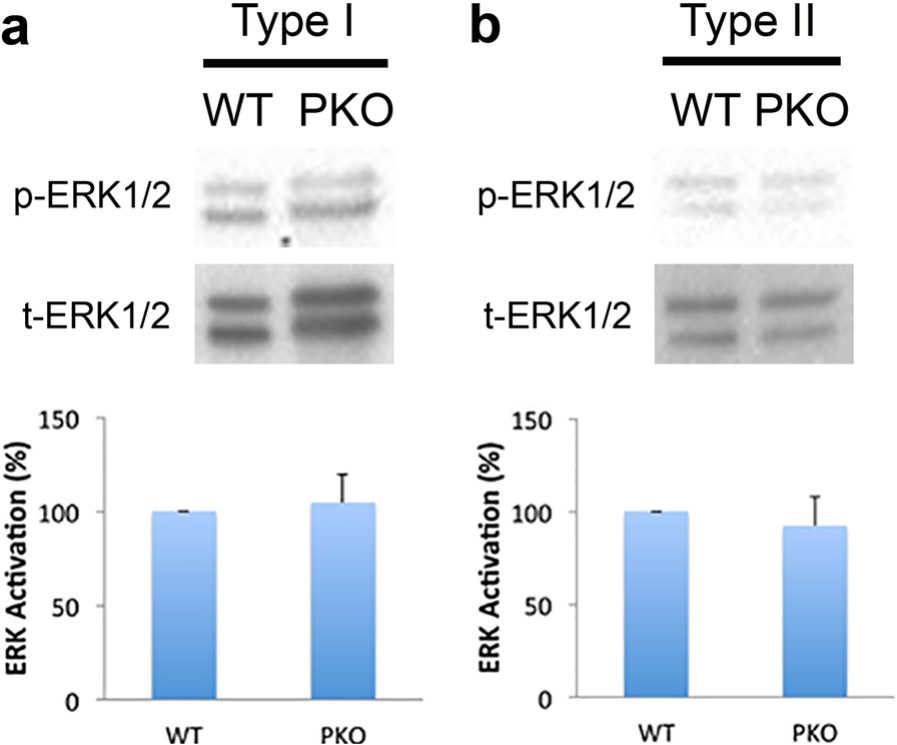
ERK1/2 activation is not responsible for the enhanced proliferation of PKO pericytes. **a** Western blots and quantification of phosphorylated ERK1/2 (*p-ERK1/2*) expression in wild-type (*WT*) and laminin γ1^flox/flox^:Pdgfrβ-Cre^+^(*PKO*) type I pericytes. Total ERK1/2 *(t-ERK1/2*) was used as a loading control. *n* = 3. **b** Western blots and quantification of p-ERK1/2 expression in WT and PKO type II pericytes. t-ERK1/2 was used as a loading control. *n* = 3. Data are shown as mean ± SD

Accumulating evidence suggests that ERK and p38 MAPK pathways play important roles in the proliferation and differentiation of various mammalian cells [37–39]. To determine if these pathways are involved in loss-of-laminin-induced proliferation of pericytes, we examined the activation/phosphorylation of ERK1/2 and p38. Consistent with previous reports demonstrating critical roles of the ERK pathway in pericyte proliferation [40, 41], we found high levels of phosphorylated ERK1/2 in both type I and type II pericytes (Fig. 4), although no significant differences in ERK1/2 activation were observed between WT and PKO pericytes (both type I and type II) (Fig. 4). These data suggest that the ERK pathway regulates pericyte proliferation, but is not responsible for the enhanced proliferation of PKO pericytes. Additionally, we also examined the activation of p38. Negligible levels of phosphorylated p38 were detected in both type I and type II pericytes independent of their genotypes (WT vs. PKO; data not shown), suggesting that p38 may not be involved in the proliferation of type I and type II pericytes.

### Laminin inhibits adipogenesis of type I pericytes

**Fig. 5:**
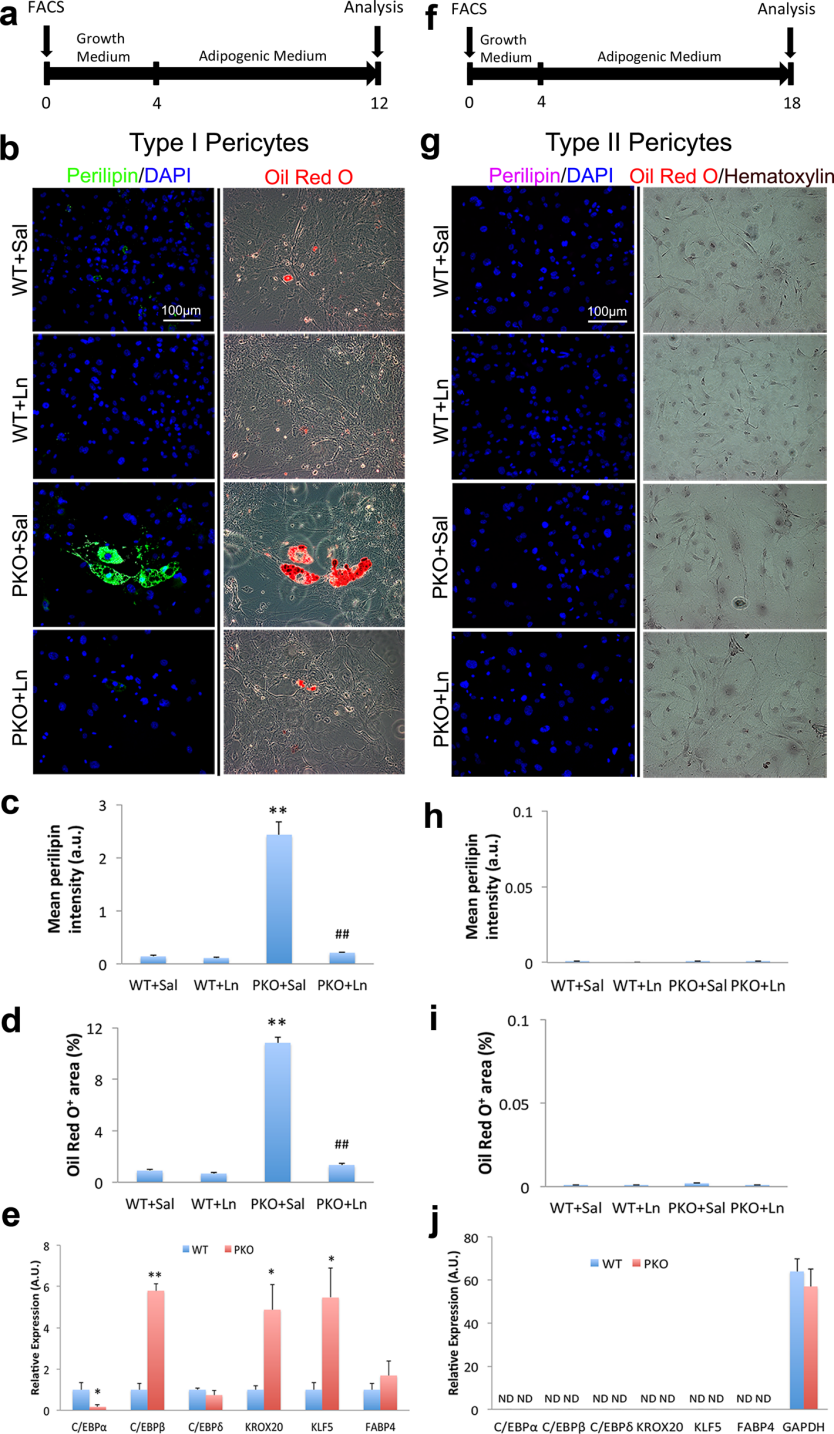
Laminin inhibits adipogenic differentiation of type I pericytes. **a** Diagram of experimental design for adipogenic differentiation of type I pericytes. **b** Immunocytochemical analysis of perilipin (*green*) and Oil red O (*red*) staining in wild-type (*WT*) and laminin γ1^flox/flox^:Pdgfrβ-Cre^+^(*PKO*) type I pericytes in the presence of saline (*Sal*) or laminin-111 (*Ln*). **c** Quantification of perilipin (*green*) expression in type I pericytes after adipogenic differentiation. *n* = 4. **d** Percentage of Oil Red O^+^ (*red*) area in type I pericytes after adipogenic differentiation. *n* = 4. **e** Quantitative RT-PCR analyses of C/EBPα, C/EBPβ, C/EBPδ, KROX20, KLF5, and FABP4 expression in type I pericytes 4 days after adipogenic differentiation. *n* = 4. **f** Diagram of experimental design for long-term adipogenic differentiation of type II pericytes. **g** Immunocytochemical analysis of perilipin (*magenta*) and Oil red O (*red*)/hematoxylin (*brown*) staining in WT and PKO type II pericytes in the presence of sal or Ln. **h** Quantification of perilipin (*magenta*) expression in type II pericytes after adipogenic differentiation. *n* = 4. **i** Percentage of Oil Red O^+^ (*red*) area in type II pericytes. *n* = 4. **j** Quantitative RT-PCR analyses of C/EBPα, C/EBPβ, C/EBPδ, KROX20, KLF5, and FABP4 expression in type II pericytes 4 days after adipogenic differentiation. *n* = 4. Data are shown as mean ± SD. *p < 0.05, **p < 0.01 versus WT or WT + Sal; ^##^ *p* < 0.01 versus PKO + Sal. *Scale bars* = 100 μm

**Fig. 6:**
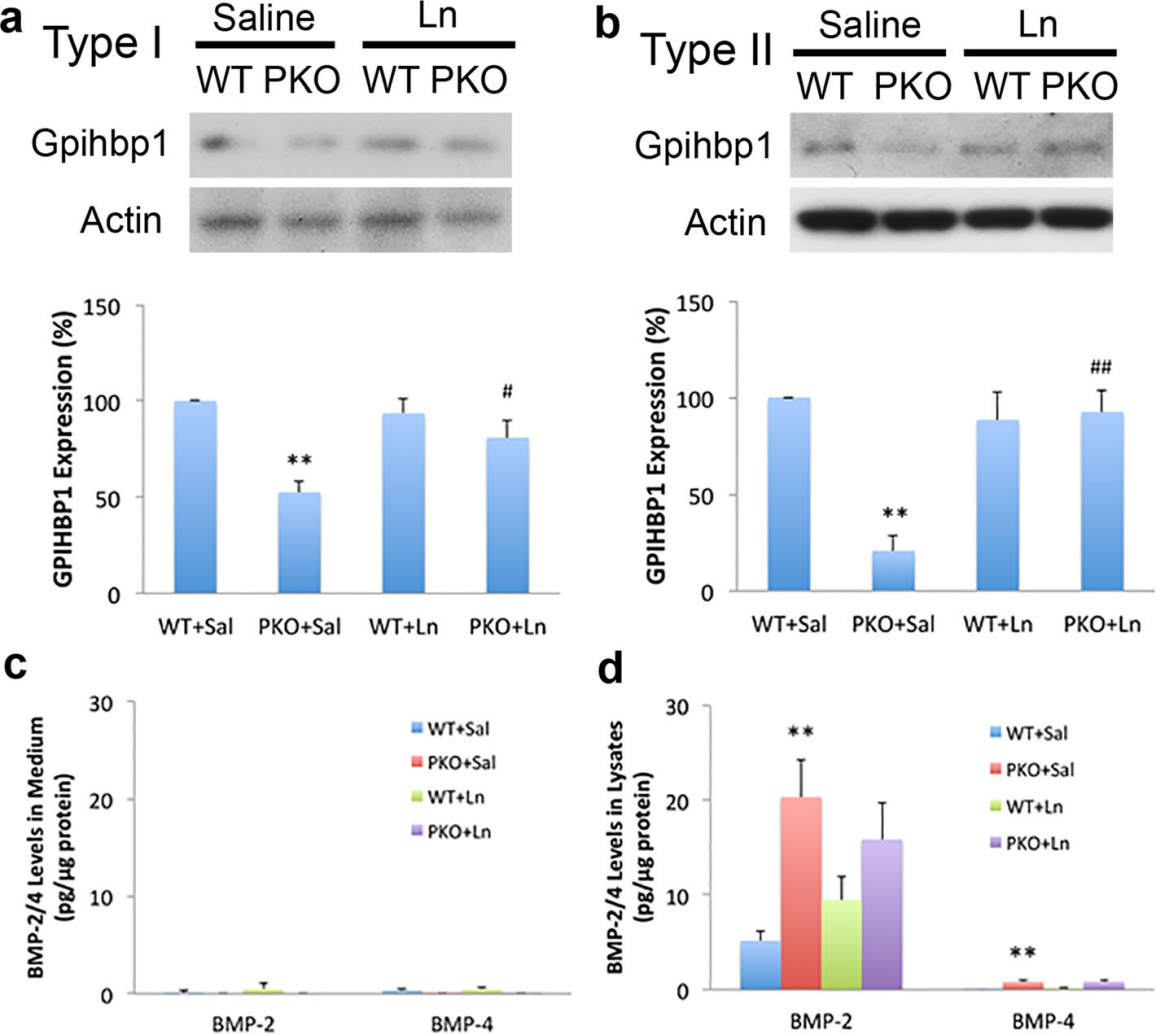
Gpihbp1 and BMP-2/4 expression in subpopulations of pericytes. **a** Western blots and quantification of gpihbp1 expression in wild-type (*WT*) and laminin γ1^flox/flox^:Pdgfrβ-Cre^+^(*PKO*) type I pericytes in the presence of saline (*Sal*) or laminin-111 (*Ln*). Actin was used as a loading control. *n* = 4. **b** Western blots and quantification of gpihbp1 expression in WT and PKO type II pericytes in the presence of Sal or Ln. Actin was used as a loading control. *n* = 4. **c** ELISA analyses of BMP-2 and BMP-4 levels in conditioned medium from type I pericytes after adipogenic differentiation for 8 days. *n* = 3. **d** ELISA analyses of BMP-2 and BMP-4 levels in cell lysates from type I pericytes after adipogenic differentiation for 8 days. *n* = 3. Data are shown as mean ± SD. **p < 0.01 versus WT + Sal; ^#^ *p* < 0.05, ^##^ *p* < 0.01 versus PKO + Sal

Accumulating evidence suggests that type I rather than type II pericytes are adipogenic [14, 23, 25]. To investigate the role of laminin in pericyte adipogenesis, we isolated type I and type II pericytes from WT and PKO muscles and examined their adipogenic differentiation in vitro (Fig. 5a). After 8 days in adipogenic medium, few WT type I pericytes expressed the adipocyte marker perilipin or were stained by Oil Red O, a dye that specifically labels lipids in adipocytes (Fig. 5b). PKO type I pericytes, however, showed features of mature adipocytes at both the morphological and biochemical levels. Cells with typical adipocyte morphology (large cells with multiple vacuoles) that were positive for perilipin and Oil Red O were observed (Fig. 5b). Quantifications revealed significantly higher levels of perilipin intensity (Fig. 5c) and Oil Red O^+^ area (Fig. 5d) in PKO type I pericytes, suggesting that laminin inhibits the adipogenic differentiation of type I pericytes. Furthermore, we found that the adipogenic differentiation of PKO type I pericytes was dramatically diminished in the presence of exogenous laminin (Fig. 5b–d), suggesting an anti-adipogenic potential of laminin. These data are consistent with previous studies demonstrating anti-adipogenic and beneficial roles of exogenous laminin in muscular dystrophy [21, 42–45]. To explore the molecular mechanism underlying the inhibitory effect of laminin in the adipogenesis of type I pericytes, we first examined the expression of gpihbp1, which has been shown to be involved in pericyte adipogenesis [21]. A significantly lower level of gpihbp1 was found in PKO type I pericytes, and exogenous laminin was able to increase gpihbp1 expression in type I PKO pericytes (Fig. 6a), suggesting that gpihbp1 may regulate the adipogenesis of type I pericytes. Next, we further examined the expression of various adipogenic factors, including C/EBPα, C/EBPβ, C/EBPδ, KROX20, KLF5, and FABP4, in type I pericytes by quantitative RT-PCR. Compared to WT type I pericytes, PKO type I pericytes demonstrated a dramatic (four- to six-fold) increase in the expression of C/EBPβ, KROX20, and KLF5, and a slight decrease of C/EBPα 4 days after adipogenic differentiation (Fig. 5e). These data suggest that laminin negatively regulates adipogenesis of type I pericytes via inhibiting the expression of C/EBPβ, KROX20, and KLF5.

Accumulating evidence shows that BMP-2 and BMP-4 promote adipogenic differentiation of various types of progenitor/stem cells, including mesenchymal precursor cells [46–50]. To investigate if loss-of-laminin-induced adipogenic differentiation of type I pericytes is driven by BMP proteins, we examined BMP-2 and BMP-4 levels from type I pericytes 8 days after adipogenic differentiation. Minimal levels of BMP-2 and BMP-4 were detected in conditioned medium independent of genotypes (WT vs. PKO) and environment (with vs. without exogenous laminin) (Fig. 6c), suggesting that either these proteins were not secreted by type I pericytes or were mobilized to cell surface after secretion. Thus, we further examined BMP-2 and BMP-4 levels in cell lysates from type I pericytes. Compared to WT cells, PKO type I pericytes expressed significantly higher levels of BMP-2 and BMP-4 (Fig. 6d). It should be noted, however, that the absolute level of BMP-2 is substantially higher than that of BMP-4 (Fig. 6d), suggesting that BMP-2 may play a more important role than BMP-4. In addition, exogenous laminin slightly reduced BMP-2 expression in type I PKO, although this change was not statistically significant (Fig. 6d). Laminin treatment also failed to affect BMP-4 expression in type I PKO pericytes (Fig. 6d). Together, these results suggest that both BMP proteins, especially BMP-2, may mediate loss-of-laminin-induced adipogenic differentiation of type I pericytes.

In sharp contrast to type I pericytes, type II pericytes failed to show signs of adipogenesis after 8 days in adipogenic medium (data not shown). To determine if this is due to a relatively short differentiation time, we allowed type II pericytes to differentiate in adipogenic condition for up to 14 days (Fig. 5f). Neither perilipin expression (Fig. 5g and h) nor Oil Red O signal (Fig. 5g and i) was observed in type II pericytes, independent of their genotypes (WT vs. PKO). In addition, the same results were observed in the presence of exogenous laminin (Fig. 5g–i). Consistent with these results, quantitative RT-PCR failed to detect any adipogenic factors described above in type II pericytes (Fig. 5j). Altogether, these data strongly suggest that type II pericytes are not adipogenic.

### Laminin is indispensable for myogenesis of type II pericytes

**Fig. 7:**
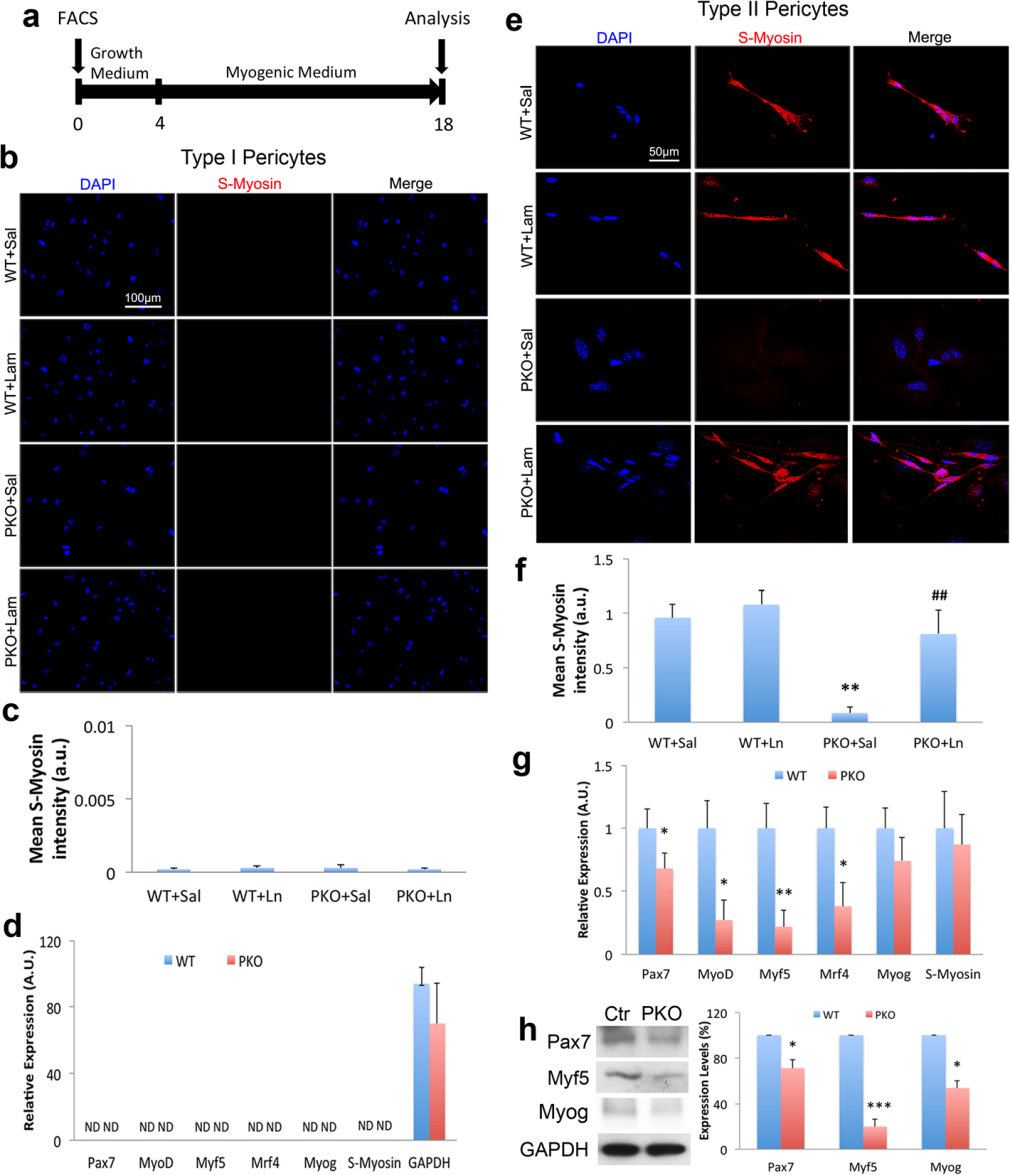
Laminin promotes myogenic differentiation of type II pericytes. **a** Diagram of experimental design for myogenic differentiation. **b** Immunocytochemical analysis of S-Myosin (*red*) in wild-type (*WT*) and laminin γ1^flox/flox^:Pdgfrβ-Cre^+^(*PKO*) type I pericytes in the presence of saline (*Sal*) or laminin-111 (*Ln*). **c** Quantification of S-Myosin (*red*) expression in type I pericytes after myogenic differentiation. *n* = 4. **d** Quantitative RT-PCR analyses of Pax7, MyoD, Myf5, Mrf4, myogenin (*Myog*), and S-Myosin expression in type I pericytes 4 days after myogenic differentiation. *n* = 4. **e** Immunocytochemical analysis of S-Myosin (*red*) in WT and PKO type II pericytes in the presence of sal or Ln. **f** Quantification of S-Myosin (*red*) expression in type II pericytes after myogenic differentiation. *n* = 4. **p < 0.01 versus WT + Sal. **g** Quantitative RT-PCR analyses of Pax7, MyoD, Myf5, Mrf4, Myog, and S-Myosin expression in type II pericytes 4 days after myogenic differentiation. *n* = 4. **h** Western blots and quantification of Pax7, Myf5, and Myog expression in WT and PKO type II pericytes 7 days after myogenic differentiation. GAPDH was used as a loading control. *n* = 4–5. Data are shown as mean ± SD. ^#^ *p* < 0.05 versus PKO + Sal; *p < 0.05, **p < 0.01, ***p < 0.001 versus WT. *Scale bars* = 100 μm in **b** and 50 μm in **d**

It has been shown that type II, but not type I, pericytes have myogenic potential and are able to differentiate into myogenic cells to repair muscle injury [14, 23, 25]. To determine the role of laminin in pericyte myogenesis, we isolated type I and type II pericytes from WT and PKO muscles using FACS, as described above, and examined their myogenic ability in vitro (Fig. 7a). Consistent with previous reports that type II rather than type I pericytes are myogenic [14, 23, 25], we failed to detect sarcomere myosin (S-Myosin), a marker for mature myotubes/myofibers, in type I pericytes after 14 days in myogenic medium regardless of their genotypes (WT vs. PKO) or environment (with vs. without exogenous laminin) (Fig. 7b). Quantification from four independent experiments revealed negligible levels of S-Myosin expression in type I pericytes (Fig. 7c). Similarly, quantitative RT-PCR failed to detect any myogenic factors, including Pax7, MyoD, Myf5, Mrf4, Myog, and S-Myosin, in type I pericytes 4 days after myogenic differentiation (Fig. 7d). These data strongly indicate that type I pericytes are unable to undergo myogenic differentiation.

Next, we performed in vitro myogenic assay using type II pericytes isolated from WT and PKO muscles. After 14 days in myogenic medium, type II pericytes isolated from WT mice aggregated together, forming myotubes that were S-Myosin-positive (Fig. 7e). Type II pericytes isolated from PKO mice, on the other hand, failed to express S-Myosin or form myotube-like structures after differentiation for 14 days (Fig. 7e). Interestingly, exogenous laminin successfully induced the formation of S-Myosin^+^ myotubes in PKO type II pericytes without affecting WT type II pericytes (Fig. 7e). Quantification revealed a significant reduction of S-Myosin expression in PKO type II pericytes compared to WT cells, and a substantial increase of S-Myosin expression in PKO type II pericytes treated with exogenous laminin compared to those treated with saline (Fig. 7f). In addition, we also examined the expression of Myogenin (Myog), a key transcription factor indispensable for myogenesis. Compared to WT type II pericytes, PKO type II pericytes demonstrated significantly lower levels of Myog (Additional file 1: Figure S3). Additionally, exogenous laminin successfully enhanced the expression of Myog in PKO type II pericytes without affecting that in WT cells (Additional file 1: Figure S3). These results suggest that laminin is indispensable for the myogenic differentiation of type II pericytes.

To explore the molecular mechanism underlying the role of laminin in the myogenesis of type II pericytes, we first examined the expression of gpihbp1, which has been shown to regulate pericyte myogenesis [21]. Compared to WT type II pericytes, PKO type II pericytes showed reduced expression of gpihbp1 (Fig. 6b). Additionally, exogenous laminin was able to increase gpihbp1 expression in PKO type II pericytes (Fig. 6b), suggesting that myogenesis of type II pericytes may be mediated by gpihbp1. Next, we examined the expression of various myogenic factors in type II pericytes by quantitative RT-PCR. Compared to WT controls, PKO type II pericytes demonstrated a significant reduction in the expression of Pax7, MyoD, Myf5, and Mrf4 4 days after myogenic differentiation (Fig. 7g). In addition, we further examined the expression of Pax7, Myf5, and Myog in type II pericytes at protein level 7 days after myogenic differentiation. Western blot analyses revealed a 30% and 80% reduction of Pax7 and Myf5 in PKO type II pericytes, respectively, compared to WT controls (Fig. 7h). Although Myog mRNA was not altered at 4 days after myogenic differentiation (Fig. 7g), its protein level was dramatically reduced in PKO type II pericytes by 7 (Fig. 7h) and 14 (Additional file 1: Figure S3) days after myogenic differentiation. Furthermore, we also examined the activation of the p38 MAPK pathway, which plays a crucial role in the myogenesis of satellite cells and/or myoblasts [51–55]. Negligible levels of phosphorylated p38 were detected in type II pericytes independent of their genotypes (WT vs. PKO; data not shown), suggesting that the p38 MAPK pathway plays a minimal role in myogenic differentiation of type II pericytes. Together, these data suggest that: (1) laminin induces myogenesis of type II pericytes via activating various myogenic transcription factors; and (2) laminin is involved in the differentiation of pericytes into Pax7^+^, Myf5^+^, and Myog^+^ myogenic cells during myogenesis, which is consistent with previous studies showing that muscle regeneration fails when Pax7 expression is genetically ablated [8, 56, 57].

**Fig. 8:**
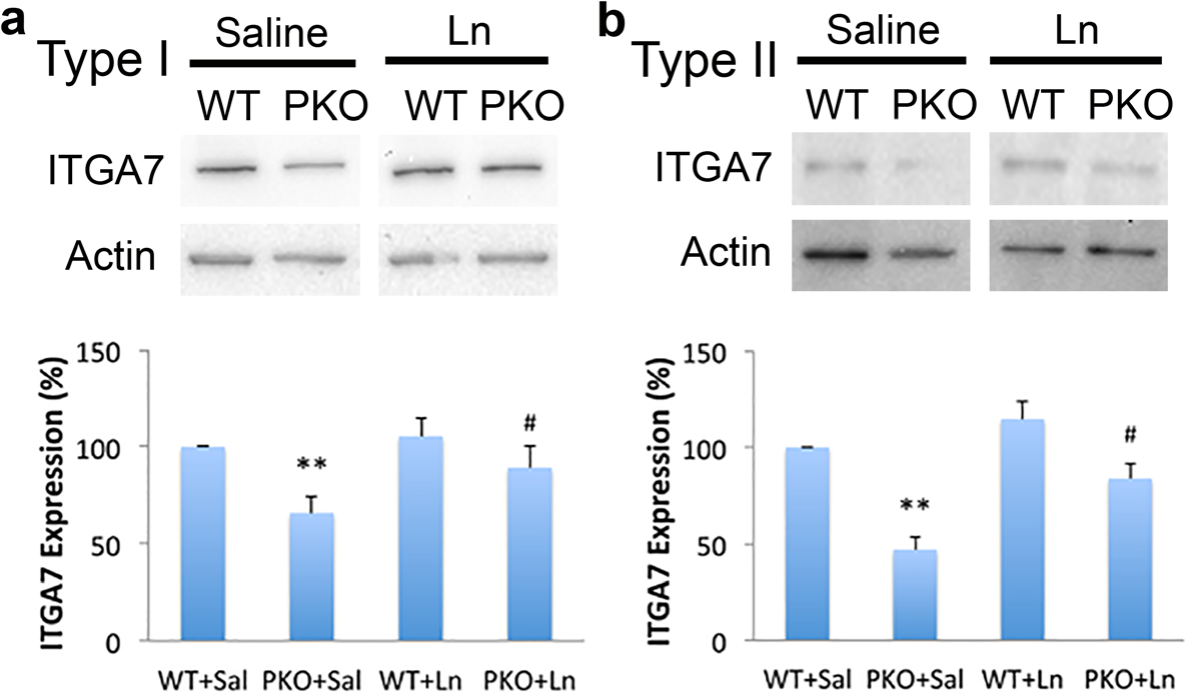
Laminin regulates ITGA7 expression in both type I and type II pericytes. **a** Western blots and quantification of integrin α7 (*ITGA7*; 120 kDa) expression in wild-type (*WT*) and laminin γ1^flox/flox^:Pdgfrβ-Cre^+^(*PKO*) type I pericytes in the presence of saline (*Sal*) or laminin-111 (*Ln*). Actin was used as a loading control. *n* = 3. **b** Western blots and quantification of ITGA7 (120 kDa) expression in WT and PKO type II pericytes in the presence of Sal or Ln. Actin was used as a loading control. *n* = 3. Data are shown as mean ± SD. **p < 0.01 versus WT + Sal; ^#^ *p* < 0.05 versus PKO + Sal

It has been shown that integrin α7 (ITGA7), a molecular marker for myoblasts, functions as a laminin receptor. To examine whether the differences in myogenic differentiation between type I and type II pericytes in the presence or absence of exogenous laminin are due to abnormal expression of ITGA7, we performed Western blot analysis. Consistent with a previous study reporting *ITGA7* mRNA transcript in pericytes/MSCs [58], ITGA7 protein (120 kDa) was detected in both type I and type II pericytes (Fig. 8). Compared to WT controls, substantially lower levels of ITGA7 were found in PKO pericytes (both type I and type II) (Fig. 8), suggesting that laminin level positively correlates with ITGA7 expression in pericytes. In addition, exogenous laminin dramatically increased ITGA7 expression in PKO pericytes (both type I and type II) without affecting that in WT pericytes (Fig. 8), again suggesting that laminin may regulate ITGA7 expression in pericytes. Since ITGA7 is expressed in both type I and type II pericytes, it is less likely to be responsible for their different myogenic potential.

## Discussion

Consistent with previous reports [14, 23, 25], we showed that type I pericytes are adipogenic and type II pericytes are myogenic under in vitro conditions. We also demonstrated that loss of laminin increases the proliferation of both type I and type II pericytes in vitro, but preferentially induces the proliferation of type II pericytes in vivo. In addition, lack of laminin also promotes adipogenic differentiation of type I pericytes and inhibits myogenic differentiation of type II pericytes in vitro. These results are consistent with: (1) the observation that both type I and type II pericytes have direct contact with laminin-rich basement membrane under physiological conditions; (2) the “inhibitory” effect of laminin on pericyte differentiation in the brain [59]; and (3) our previous report [21] that loss of laminin in PDGFRβ^+^ pericytes (both type I and type II) increases their proliferation, promotes their adipogenic differentiation, and diminishes their myogenic differentiation. Together, our data suggest that laminin actively but differentially regulates the proliferation and differentiation of type I and type II pericytes. The mechanism linking laminin and different responses of type I and type II pericytes, however, remains not fully understood. Our mechanistic studies showed that: (1) loss of laminin activates the adipogenic program in type I pericytes by enhancing the expression of various adipogenic factors, including C/EBPβ, KROX20, and KLF5; and (2) loss of laminin inactivates the myogenic program in type II pericytes by decreasing the expression of almost all myogenic transcription factors, including Pax7, MyoD, Myf5, Mrf4, and Myog. These data suggest that the intrinsic difference between type I and type II pericytes may be responsible for their distinct behaviors to loss of laminin. Consistent with previous studies [14, 60], adipogenic factors were detected in type I but not type II pericytes after adipogenic differentiation, whereas myogenic factors were found in type II but not type I pericytes after myogenic differentiation. These results support that type I and type II pericytes are intrinsically different and have distinct differentiation programs. Altogether, our data suggest that laminin differentially regulates the differentiation and fate determination of type I and type II pericytes by acting on distinct transcription factors in these cells.

It should be noted that Nestin-GFP^+^ myofibers are not detected in Nestin-GFP transgenic mice in vivo, although type II pericytes show myogenic activity in vitro. This may be due to the following three possibilities. First, type II pericytes may lose Nestin-GFP expression after myogenic differentiation, generating GFP-negative myofibers. Second, the contribution of pericytes to myogenesis in vivo may be below the detection level under physiological conditions. Third, pericytes do not have myogenic capability in vivo, but obtain myogenic potential in vitro. Recently, it has been shown that transplantation of DsRed-expressing type II pericytes into injured muscles leads to DsRed-positive myofibers after 2 weeks [14], suggesting that type II pericytes are able to undergo myogenic differentiation in vivo, at least under pathological conditions. Therefore, we favor the first two possibilities.

In the in vitro proliferation assay, we showed that exogenous laminin failed to decrease the proliferation of WT pericytes. This could be due to the fact that endogenous laminin produced by WT pericytes is sufficient to inhibit and maintain the proliferation at baseline. An alternative explanation is that endogenous pericytic laminin and exogenous laminin may be different in their compositions and functions. The exogenous laminin we used in this study is mouse laminin-111, which is the only commercially available recombinant laminin of mouse origin. Although expressed at the embryonic stage, this laminin isoform is absent in adult skeletal muscles [61–67]. In addition, although the exact laminin isoforms produced by pericytes are not clear, many other laminin chains, including laminin α4 and β2, are detected [68]. Ongoing projects in our laboratory focus on identifying laminin isoforms synthesized by pericytes and investigating their biological functions.

The fact that exogenous laminin successfully reversed the proliferation and differentiation phenotypes of PKO pericytes suggests that laminin-111 could at least partially compensate for the loss of pericyte-derived laminin isoforms. This is consistent with previous reports on the compensatory effects of laminin-111. For example, expressing laminin α1 is able to improve the general health (body weight and lifespan) and muscle deficit in laminin α2-deficient mice [69–74], a widely used congenital muscular dystrophy model [75–77]. Furthermore, recombinant laminin-111 has been shown to improve muscle pathology and function in various muscular dystrophy models, including those caused by lack of laminin α2 [42, 44] and laminin γ1 [21]. Additionally, laminin α1, especially its globular domains, has been shown to partially compensate for the developmental defect caused by loss of laminin α5 [78, 79]. Given the diverse biological functions and strong therapeutic potential of laminin-111, it is not surprising to see that the deficits in proliferation and differentiation of PKO pericytes can be rescued by exogenous laminin-111.

In muscle injury induced by glycerol or BaCl_2_, both type I and type II pericytes were reported to expand in vivo [14]. In contrast to that study, we found that type II pericytes predominantly proliferate in pericytic laminin-deficient mice. Given that type II pericytes have myogenic capability [14] and that the pericytic laminin-deficient mutants develop severe muscular dystrophy [21], this finding suggests that laminin is indispensable for the myogenesis of type II pericytes. The discrepancy may be explained by either pericytic laminin only regulates the proliferation of type II pericytes, or different injury models create different microenvironments that lead to distinct outcomes. Given that loss of pericytic laminin enhances the proliferation of both type I and type II pericytes in vitro, we favor the second possibility. The exact microenvironment created in PKO mice and how it induces predominant proliferation of type II pericytes in vivo, however, remain unclear. Answers to these questions would allow us to identify subtype-specific pathways, separate subtype-specific functions, and hopefully achieve therapeutic potential by manipulating different subtypes of pericytes.

## Conclusions

Laminin predominantly inhibits the proliferation of type II pericytes in vivo, and negatively and positively regulates the adipogenic and myogenic differentiation of type I and type II pericytes, respectively.

## Additional file

Additional file 1:
**Figure S1.** Representative dot plots for PDGFRβ-PE and Nestin-GFP in FACS analysis. (a) Representative dot plot for PDGFRβ-FMO control. (b) Representative dot plot showing sorting gates. **Figure S2.** Laminin inhibits the proliferation of type I and type II pericytes in vitro. (a) Quantification of pH3^+^ cell percentage in type I pericytes in the presence of sal or ln. *n* = 4. (b) Quantification of pH3^+^ cell percentage in type II pericytes in the presence of Sal or Ln. *n* = 4. *Ln* laminin-111, *Sal* saline, *WT* wildtype. Data are shown as mean ± SD. ***p* < 0.01 versus WT + Sal; ^##^ *p* < 0.01 versus PKO + Sal. **Figure S3.** Laminin induces Myogenin expression in type II pericytes after myogenic differentiation. Western blots and quantification of Myog expression in WT and PKO type II pericytes after myogenic differentiation. Actin was used as a loading control. *n* = 4. *Ln* laminin-111, *Sal* saline, *WT* wildtype. Data are shown as mean ± SD. ***p* < 0.01 versus WT + Sal; ^#^ *p* < 0.05 versus PKO + Sal. (DOCX 17413 kb)

## Abbreviations

F/F: Laminin γ1^flox/flox^
FAP: Fibro/adipogenic progenitor
FMO: Fluorescence minus one
ITGA7: Integrin α7
MSC: Mesenchymal stem cell
Myog: Myogenin
pH3: Phospho-Histone H3
PIC: PW1^+^/Pax7^–^ interstitial cell
PKO: Laminin γ1^flox/flox^:Pdgfrβ-Cre^+^
WT: Wild-type
