# Supplementary material for: Laminin differentially regulates the stemness of type I and type II pericytes
Source: Stem Cell Res Ther. 2017 Feb 7;8:28. doi: 10.1186/s13287-017-0479-4 (PMC5297126; doi:10.1186/s13287-017-0479-4)
Supplement: Additional file 1: — Figure S1. Representative dot plots for PDGFRβ-PE and Nestin-GFP in FACS analysis. (a) Representative dot plot for PDGFRβ-FMO control. (b) Representative dot plot showing sorting gates. Figure S2. Laminin inhibits the proliferation of type I and type II pericytes in vitro. (a) Quantification of pH3+ cell percentage in type I pericytes in the presence of sal or ln. n = 4. (b) Quantification of pH3+ cell percentage in type II pericytes in the presence of Sal or Ln. n = 4. Ln laminin-111, Sal saline, WT wildtype. Data are shown as mean ± SD. **p < 0.01 versus WT + Sal; ## p < 0.01 versus PKO + Sal. Figure S3. Laminin induces Myogenin expression in type II pericytes after myogenic differentiation. Western blots and quantification of Myog expression in WT and PKO type II pericytes after myogenic differentiation. Actin was used as a loading control. n = 4. Ln laminin-111, Sal saline, WT wildtype. Data are shown as mean ± SD. **p < 0.01 versus WT + Sal; # p < 0.05 versus PKO + Sal. (DOCX 17413 kb) [file 13287_2017_479_MOESM1_ESM.docx]

Laminin differentially regulates the stemness of type I and type II pericytes

Jyoti Gautam, Abhijit Nirwane, and Yao Yao^*^


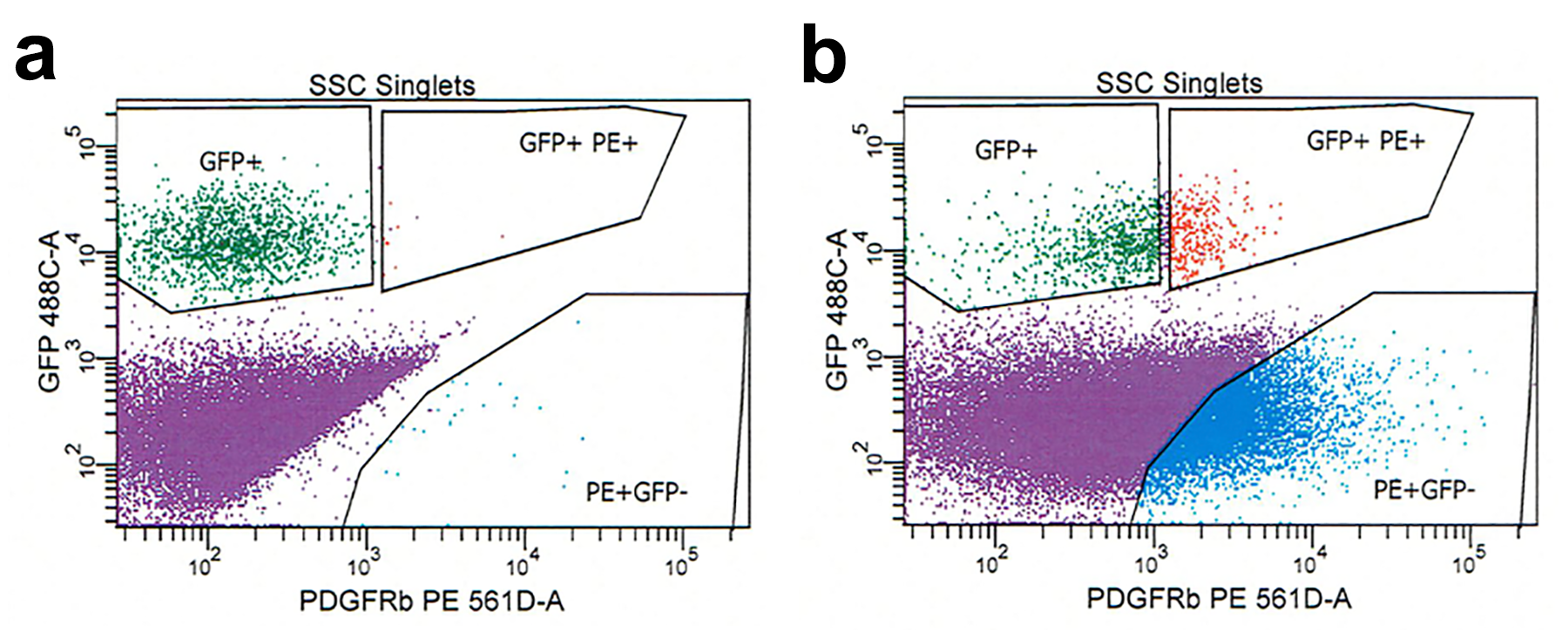


Supplementary Figure 1. Representative dot plots for PDGFRβ-PE and Nestin-GFP in FACS analysis. (**a**) Representative dot plot for PDGFRβ-FMO control. (**b**) Representative dot plot showing sorting gates.


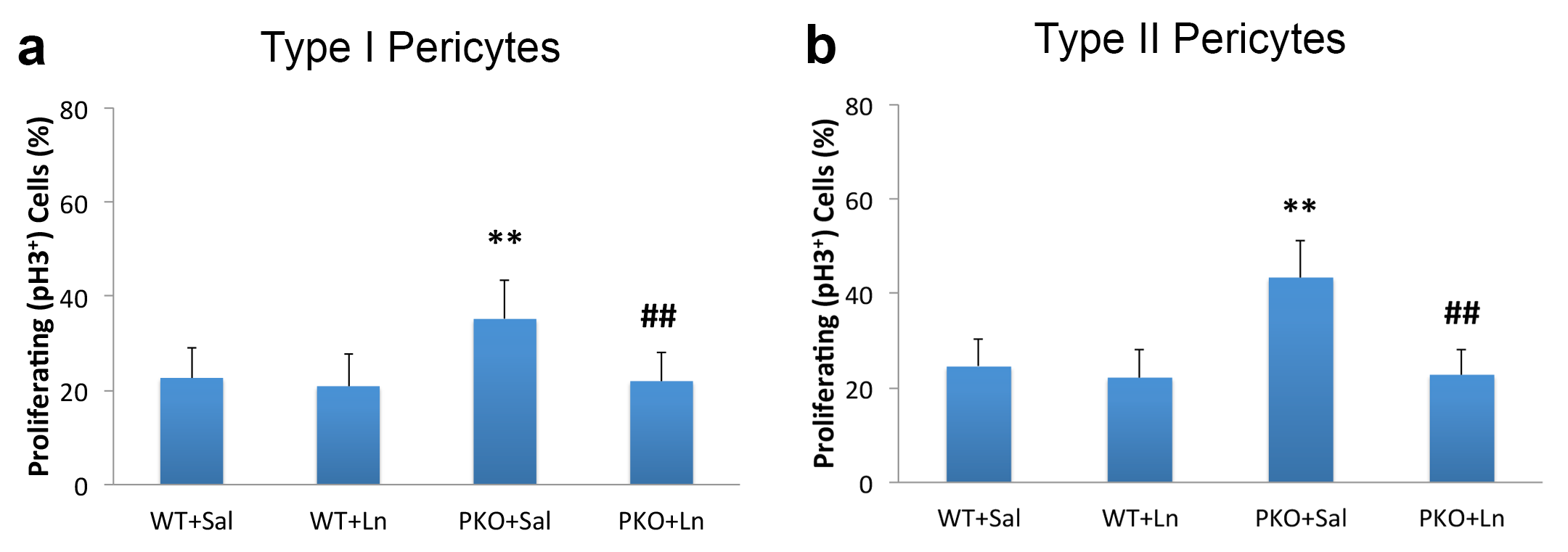


Supplementary Figure 2. Laminin inhibits the proliferation of type I and type II pericytes. (**a**) Quantification of pH3^+^ cell percentage in type I pericytes in the presence of Sal or Ln. n=4. (**b**) Quantification of pH3^+^ cell percentage in type II pericytes in the presence of Sal or Ln. n=4. WT, wildtype; Sal, saline; Ln, laminin-111. Data are shown as mean ± S.D.. **p<0.01 versus WT+Sal; ^##^p<0.01 versus PKO+Sal.


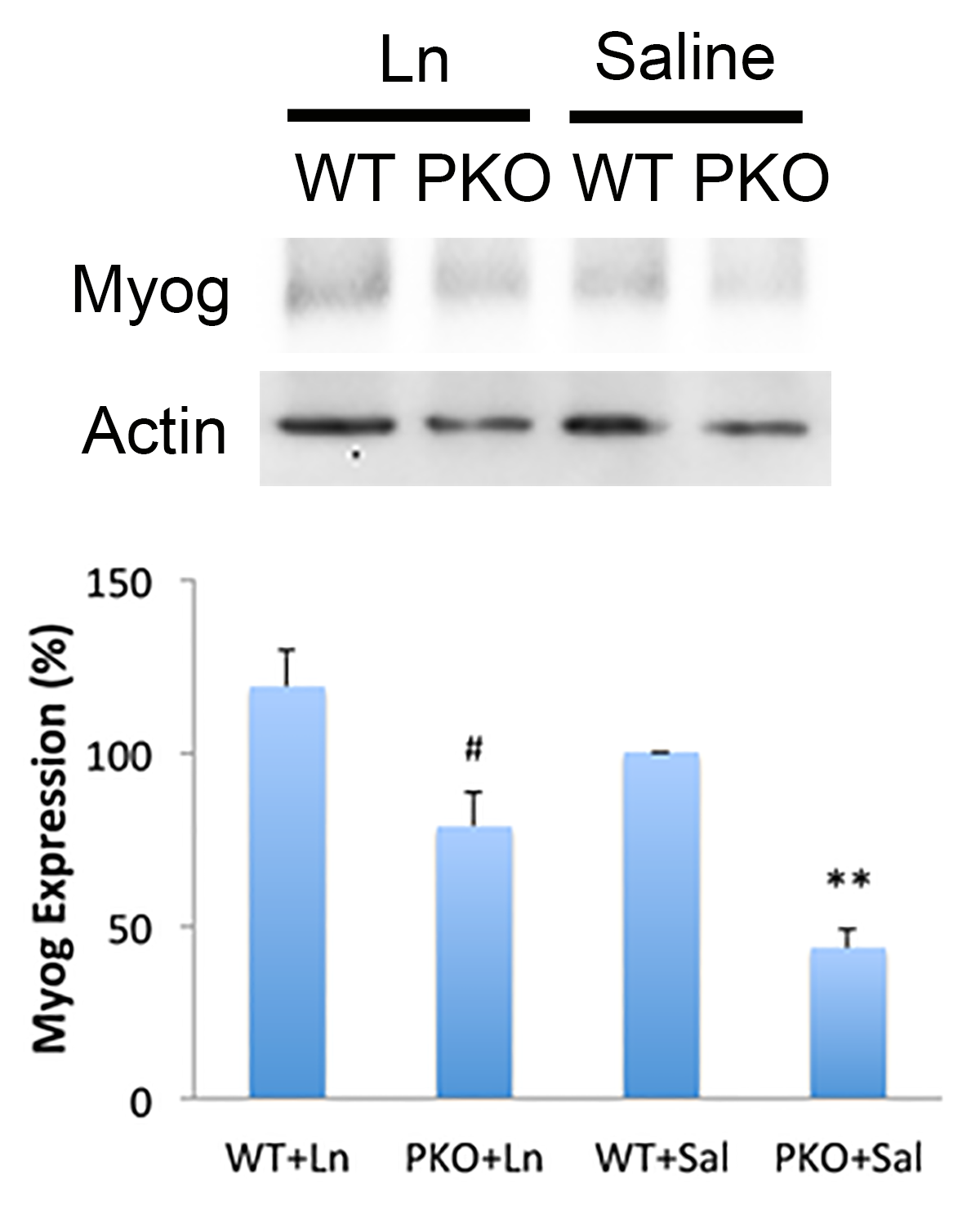


Supplementary Figure 3. Laminin induces Myogenin expression in Type II pericytes after myogenic differentiation. Western blots and quantification of Myog expression in WT and PKO type II pericytes after myogenic differentiation. Actin was used as a loading control. n=4. WT, wildtype; Sal, saline; Ln, laminin-111. Data are shown as mean ± S.D.. **p<0.01 versus WT+Sal; ^#^p<0.05 versus PKO+Sal.
